# Cyclic Fevers in Adult Diagnosed As Hyperimmunoglobulin D Syndrome

**DOI:** 10.7759/cureus.23878

**Published:** 2022-04-06

**Authors:** Merin Reji, Rupak Thapa

**Affiliations:** 1 Internal Medicine, Atrium Health Wake Forest Baptist, Winston-Salem, USA; 2 Rheumatology, Atrium Health Wake Forest Baptist, Winston-Salem, USA

**Keywords:** mevalonic aciduria, mevalonate kinase deficiency, genetic disease, hyperimmunoglobulin d syndrome, cyclical fevers

## Abstract

Hyper immunoglobulin D Syndrome (HIDS) is a rare autosomal recessive disease often presents during infancy. The disease is caused by an abnormal gene that codes for mevalonate kinase (MVK). This results in recurrent fever episodes and gastrointestinal discomfort (including diarrhea, joint pain, and oral sores). High fever is the most common symptom, occurring every few weeks to months. Patients may also have other findings, including lymphadenopathy and arthralgia. In this report, we discuss a rare diagnosis of HIDS is an adult and discuss our case in the context of existing literature. Given the nonspecific symptoms and the fact that it is often diagnosed in childhood, HIDS can be a challenging but essential diagnosis in adults with persistent, cyclical fevers.

## Introduction

Hyper immunoglobulin D syndrome (HIDS) is a sporadic genetic disorder that results in periodic fevers, nausea, vomiting, myalgia, and general malaise in patients [1]. HIDS falls under the umbrella of mevalonate kinase (MVK) deficiency, which occurs due to a mutation in the MVK gene, resulting in the deficiency of the MVK enzyme. The MVK enzyme plays a role in cholesterol and steroid production. It is theorized that its deficiency results in a build-up of mevalonate acid (which the MVK enzyme acts on), cholesterol, steroids, and bile acids [2]. The exact mechanism of why the periodic fevers occur is unclear. However, the theory is that the build-up of these molecules-which play a role in cellular functions-result in the fevers and symptomology seen in MVK deficiency [2]. However, it is known that the fever and symptoms experienced in HIDS are often brought on by episodes of inflammation such as infection, psychological stress, and injury [3,4].

MVK deficiency presents on a spectrum of severity, with HIDS as the less severe form of presentation. In HIDS, patients generally have 1% to 20% normal mevalonate kinase functioning. The more severe presentation is mevalonic aciduria (MVA), where patients generally have less than 1% of their MVK enzyme functioning normally [2,5]. Those with MVA present significant growth delay from childhood and experience ataxia, ocular disorders, and failure to thrive [2]. MVK deficiency, as a whole, is a rare condition with an estimated relevance of more than 300 people worldwide [5]. According to the National Organization for Rare Disorders (NORD), the exact incidence and relevance of MVK deficiency are not known due to possible instances of missed diagnosis of this condition and limited data on the condition [5].

In this report, we present a case of HIDS in a young woman who had been undiagnosed for over three decades. The most common symptoms of HIDS include cyclical fevers, gastrointestinal discomfort, lymphadenopathy, malaise, oral sores, and arthralgia (all of which are mainly nonspecific) [1]. In addition, patients may present with only some of these symptoms. Although the typical presentation of HIDS may vary, the German Society of Pediatric Rheumatology created a consensus in 2020 on the expected clinical phenotype of patients with mevalonate kinase deficiency and HIDS, which listed cyclic fevers greater than six months in duration with associated myalgia, oral ulceration, headache, and abdominal pain, with symptoms starting before age one [6]. Even with such criteria to meet, many diagnoses could fit this pattern, including infections, malignancy, and other conditions including Familial Mediterranean Fever, Periodic Fever, Aphthous Stomatitis, Pharyngitis, Adenitis (PFAPA) [1,6]. These diagnoses are all less common in the adult population and, therefore, less commonly considered in the differential for adult patients. The case presented in this report demonstrates this uncommon genetic disorder, the diagnostic challenges associated with identifying HIDS as an adult, and how this diagnosis brought clarity and control for the patient over her disease process.

This article was previously presented as a meeting abstract at the Virtual Annual Scientific Session of the North Carolina Chapter of the American College of Physicians on February 11, 2022.

## Case presentation

A 29-year-old female with a past medical history of hypothyroidism and recurrent fevers presented to the rheumatology clinic at her primary care physician (PCP) for evaluation of possible sarcoidosis. The patient had a history of periodic fevers of 100.5⁰ F to 102⁰ F with associated oral ulcerations, sore throat, postnasal drip, and myalgia since childhood. She had an extensive evaluation prior to the initial consultation for these symptoms. Her workup included several rounds of complete blood counts and normal metabolic panels, an ultrasound of her head and neck, which was unremarkable, and a computed tomography (CT) scan with contrast of her chest, abdomen, and pelvis that was also unremarkable. There was no lymphadenopathy or splenomegaly consistent with sarcoidosis. During her clinical course, she had also been treated with various antibiotics for these fever episodes, including trimethoprim-sulfamethoxazole, amoxicillin, and ampicillin, without any relief of symptoms. Part of her workup included an angiotensin-converting enzyme (ACE) level, which was elevated, prompting the referral by the patient’s PCP to the rheumatology clinic for evaluation for sarcoidosis. Despite the extensive workup and treatment with antibiotics, the patient continued to have cyclical fevers and associated symptoms every three to four weeks.

At the initial rheumatology consultation, the patient was between fever cycles. She worked as a fitness trainer and maintained an active lifestyle interrupted monthly by three to four days of these fever and malaise episodes. Her rheumatological review of systems was negative except for cyclical fevers and oral ulceration. It was negative for alopecia, skin changes, photosensitivity, lymphadenopathy, and sicca symptoms, including dry eyes or dry mouth. On presentation, her initial vitals were a temperature of 98 ⁰F, blood pressure of 101/63 mmHg, heart rate of 70 beats per minute, respiratory rate of 14 breaths per minute, and oxygen saturation of 100% on room air. Physical examination showed a well-appearing female in no distress with a regular cardiac, lung, musculoskeletal, neurological, and dermatological exam. Notably, she had no rashes or lymphadenopathy. A review of her laboratory work showed an average complete blood count and complete metabolic panel. The remainder of the patient’s initial laboratory evaluation is summarized in Table 1, with ACE level normalizing when rechecked nine months after it was initially checked and found to be elevated.

**Table 1:** The patient's laboratory evaluations

Laboratory Testing	Patient’s Result
Angiotensin Converting Enzyme (initially)	111 U/L (normal 16 – 85 U/L)
Angiotensin Converting Enzyme (when rechecked nine months after the prior level was obtained)	63 U/L (normal 16 – 85 U/L)
Rheumatoid Factor	<14 IU/mL (normal <14 IU/mL)
Anti-Ribonucleoprotein Antibody	Negative
Anti-Centromere Antibody	Negative
Anti-Smith Antibody	Negative
Anti-Nuclear Antibody	Negative
Anti-Double Stranded DNA Antibody	<12.3 IU/mL (normal <30 IU/mL)
Complement C3	87 mg/dl (normal 87 – 200 mg/dl)
Complement C4	35 mg/dl (normal 19 – 52 mg/dl)
Anti-Sjogren’s Syndrome A Antibody	Negative
Anti-Sjogren’s Syndrome B Antibody	Negative
C Reactive Protein	<5 mg/L (normal <5 mg/L)
Erythrocyte Sedimentation Rate	3 mm/hr (normal 0 – 15mm/hr)
Serum Immunoglobulin A	126 mg/dL (normal 87 – 352 mg/dL)
Serum Immunoglobulin D	<1.28 mg/L (normal <14.11 mg/L)

The differential diagnoses considered for the patient included herpangina, Heerfordt-Waldenström syndrome, Familial Mediterranean Fever, hyper immunoglobulin D syndrome (HIDS), Periodic Fever, Aphthous Stomatitis, Pharyngitis, Adenitis (PFAPA) syndrome. The patient was empirically started on colchicine 0.6 mg, with instructions to take one to two tablets daily, which improved her symptoms. This regimen was employed as she had previously noticed improvement with colchicine.

**Figure 1:**
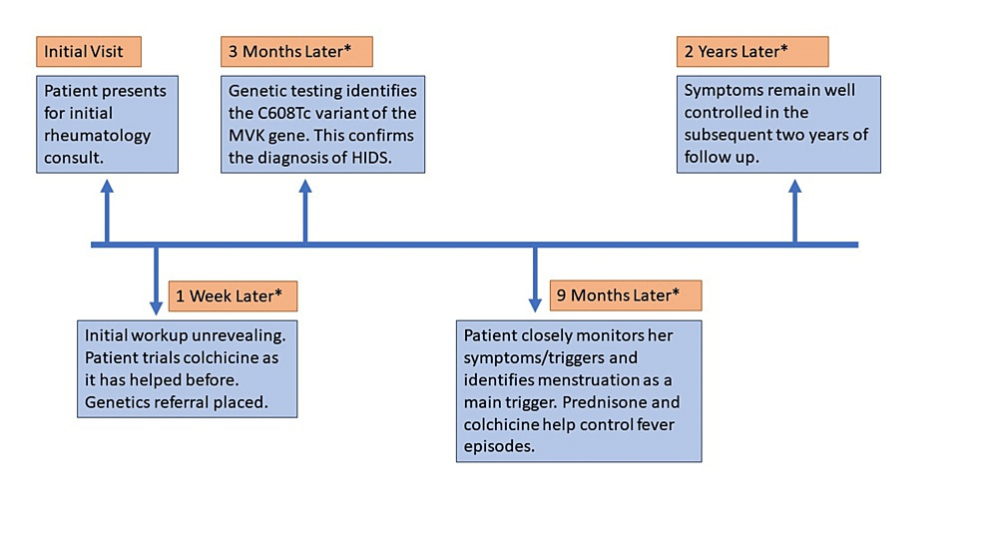
A summary of the patient’s clinical course over two years *Time references in orange boxes represent the time elapsed since the initial rheumatology consult. MVK: Mevalonate Kinase; HIDS: Hyper immunoglobulin D Syndrome

HIDS was the top differential diagnosis, and thus the patient was referred for genetic testing. The results identified the C608Tc variant of the MVK gene, which diagnosed the patient with HIDS. She reviewed her fever episodes against her overall health and identified menstruation as her most common trigger for flares. Her condition was managed with colchicine 0.6 mg daily with the dose doubled around menstruation and prednisone 5mg daily during flares. The patient chose this regimen over a more traditional route of nonsteroidal anti-inflammatory drugs (NSAIDs) and steroid taper generally used in HIDS, as colchicine significantly helped the patient’s symptom burden. The patient has been able to manage her flares on this regimen in the two years of subsequent follow-up, and her symptom burden has been significantly reduced. A summary of the patient’s clinical course is shown in Figure 1 in the form of a timeline. Interleukin-1 receptor antagonist therapy such as anakinra has been discussed with the patient for potential use in the future.

## Discussion

The constellation of symptoms experienced by the patient presented here lends itself to many diagnoses. While the patient did have an elevated ACE level of 111 U/L, which sometimes can indicate sarcoidosis, this is neither sensitive nor specific to sarcoidosis [7]. The patient’s recurrent symptoms of sore throat, myalgia, and fevers were overall not consistent with sarcoidosis. The nonspecific presentation of HIDS proves itself to be a sizable diagnostic challenge for any clinician faced with its presentation.

Diagnosing HIDS relies on a high clinical suspicion of the disease based on symptomology. This is followed by laboratory assessments which may show elevated IgD and IgA (not a requirement for diagnosis), elevated C reactive protein and erythrocyte sedimentation rate (also not always present in HIDS), and elevated urinary mevalonic acid during fever episodes [8]. Classic symptoms, elevated IgD, with or without elevated IgA, and perhaps most importantly, a high degree of clinical suspicion is sufficient to diagnose HIDS [8]. If the IgD or IgA is normal, as is in the case presented here, genetic testing can be pursued, as recommended by Mizuno et al. [8,9].

**Figure 2:**
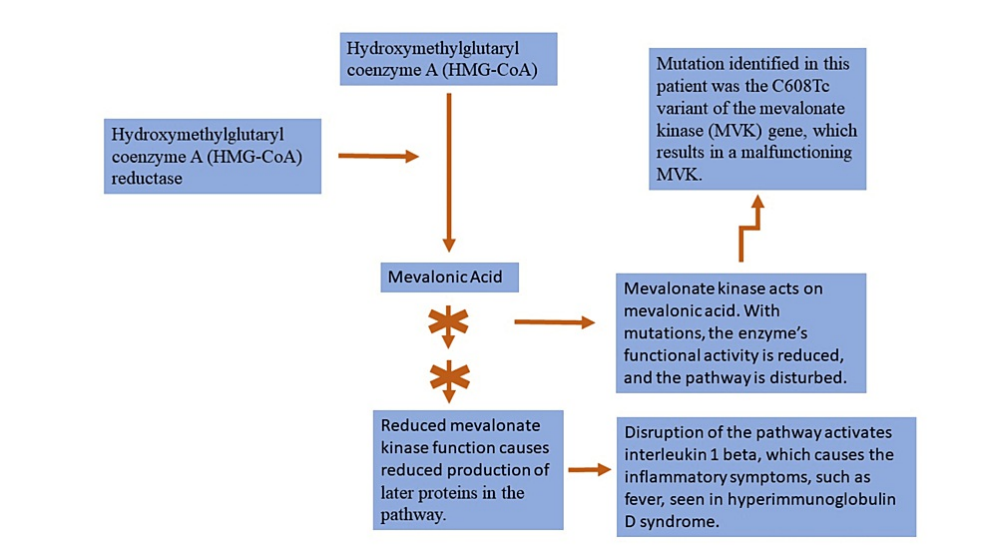
How mevalonate kinase mutations result in inflammation This figure demonstrates how a genetic mutation in the mevalonate kinase (MVK) results in the inflammatory responses seen in hyper immunoglobulin D syndrome (HIDS). In particular, disruption of the pathway shown in this figure results in reduced production of a protein called geranylgeranyl pyrophosphate, which has been linked to activating interleukin 1 beta and causing inflammation [11]. The pathway shown here is a simplistic demonstration of the critical areas affected by the mutation of the mevalonate kinase. This figure does not include all the steps involved in the steroid synthesis pathway.

The genetic testing involved in HIDS diagnosis requires further discussion. A paper by Manday et al. gives a comprehensive overview of the 63 mutations identified to this day in HIDS [10]. While the mutations are distinct, each of these mutations is a reduced function of the MVK, which disrupts the steroid synthesis pathway, as shown in Figure 2, resulting in the fever and inflammation seen in HIDS [8,11]. Specifically, disruption in the pathway shown in Figure 2 results in reduced protein production that then leads to the activation of inflammation through interleukin 1 beta [11]. Further studies have explored whether specific mutations of the MVK gene are more common in particular world regions. The 2020 study by Govindaraj et al. has noted that specific genetic mutations tend to cluster in some geographic regions [12]. This may lead to more targeted genetic screening in patients with HIDS based on their geography [12].

The recommended treatment for HIDS is an evolving field. The American College of Rheumatology recommends starting the patient on NSAIDS or a prednisone taper during fever flares and escalating to interleukin-1 receptor antagonists and tumor necrosis factor inhibitors such as anakinra, canakinumab, and etanercept in the treatment of HIDS [3]. Research is underway to identify more targeted therapies that may offer improved control over HIDS flares, focusing on anti-inflammatory agents as many patients identify triggers such as infection, stress, or trauma prior to fever episodes [3,4].

Recurrent and cyclical fevers, myalgia, and malaise can debilitate a patient’s quality of life. One study of 103 patients with HIDS internationally found that those who had six or more fever attacks in a year had a significantly lower outlook on their health and quality of life than those with fewer attacks. That study also identified the median delay to diagnosis of HIDS to be 9.9 years [13]. Therefore, this study highlights the importance of having HIDS on the differential for recurrent fevers. Early diagnosis allows patients to understand better their disease process, triggers, and treatment options to improve their quality of life. Additionally, the severe form of HIDS, known as mevalonic aciduria (MVA), can cause significant neurologic impairments [14]. Given such consequences and the hereditary pattern that may impact potential offspring, especially in patients of reproductive age, it is critical to recognize and diagnose this rare condition as early as possible.

## Conclusions

HIDS is a rare genetic disorder that can have devastating effects on patients’ quality of life. The case presented in this paper demonstrates the years of diagnostic uncertainty a patient may experience prior to reaching a definitive diagnosis. In this case, an accurate diagnosis allowed her to identify possible triggers that may be inciting her fever episodes and successfully identify menstruation as a trigger. This then further allowed her to better control her symptoms and be prepared for these fever episodes. Although rare, this case demonstrates that HIDS is an important and diagnostically challenging disorder to consider in patients with cyclical fevers.
